# Sleep Characteristics in Esport Players and Associations With Game Performance: Residual Dynamic Structural Equation Modeling

**DOI:** 10.3389/fspor.2021.697535

**Published:** 2022-01-13

**Authors:** Frode Moen, Marte Vatn, Maja Olsen, Jan Arvid Haugan, Vera Skalicka

**Affiliations:** ^1^Department of Education and Lifelong Learning, Faculty of Social and Educational Sciences, Norwegian University of Science and Technology, Trondheim, Norway; ^2^Department of Neuromedicine and Movement Science, Faculty of Medicine and Health Science, Centre for Elite Sports Research, Norwegian University of Science and Technology, Trondheim, Norway; ^3^Department of Sociology and Political Science, Faculty of Social and Educational Sciences, Norwegian University of Science and Technology, Trondheim, Norway; ^4^Department of Psychology, Faculty of Social and Educational Sciences, Norwegian University of Science and Technology, Trondheim, Norway

**Keywords:** sleep, esport, game performance, sleep staging, stress

## Abstract

The current study aimed to examine sleep characteristics of esport players and the stipulated effects of game performance on consecutive sleep characteristics using residual dynamic structural equation modeling (RDSEM). A sample of 27 Counterstrike players with a mean age of 18½ years participated in the current study. Sleep was detected over a period of 56 days with a Somnofy sleep monitor that utilizes an impulse radio ultra-wideband puls radar and Dopler technology, and weekly game performance was reported by the players. The results showed that esport players' sleep characteristics were in the lower levels of recommended guidelines and that sleep onset started later and sleep offset ended later in the morning compared with athletes from other traditional sports. The esport players displayed stable patterns in sleep onset, sleep offset, time in bed, sleep efficiency and non-REM respiration rates per minute (NREM RPM). On the between-person level, esport players with better game performance spent more time sleeping (*r* = 0.55) and scored lower on NREM RPM (*r* = −0.44). Unstandardized within-person cross-lagged paths showed that better game performance predicted subsequent earlier sleep offset. The within-level standardized estimates of the cross-lagged paths revealed that participants with better game performance spent subsequently more time in deep sleep (0.20), less time in light sleep (−0.14), less time in bed (−0.16), and displayed lower NREM RPM (−0.21), earlier sleep offset (−0.21), and onset (−0.09). The findings of better game performance being related to better sleep are discussed in terms of existing knowledge on how stress responses elicitated by poor performance might impact on non-REM respiration rates and sleep.

## Introduction

Esport is an organized and competitive way of playing video games, established by international ranking systems regulated by official leagues (Pedraza-Ramirez et al., 2020). One of the most popular games is first-person shooter (FPS) games, where the players are typically seated in front of a screen where they have to handle the movements and decisions of a virtual avatar through a controller (Sainz et al., 2020). Esport gaming involves several mental skills (Taylor, 2012; Bonnar et al., 2019; Martin-Niedecken and Schättin, 2020), most importantly perceptual cognitive skills (e.g., selective attention, inhibition, and working memory) (Pedraza-Ramirez et al., 2020) and strategic thinking (Martin-Niedecken and Schättin, 2020), as well as fine motor skills (Bonnar et al., 2019). Therefore, esport players are often referred to as “cognitive athletes” (Martin-Niedecken and Schättin, 2020).

Because of the high cognitive demands and the continuous evaluation of esport players' game performances, e.g., through gaming statistics that influence the players' rankings in the global ranking system, players are likely to be exposed to high stress loads (Taylor, 2012; Pedraza-Ramirez et al., 2020). Stress is a response to a stressor and several internal and external stressors have been identified among elite competitive esport players (Fletcher et al., 2006; Smith et al., 2019). Typical internal stressors are related to in-game communication, criticism, self-evaluations, and lack of confidence, while external stressors are related to external evaluations (ranking systems), event audience and media (Smith et al., 2019). Game results can also be considered as a potential stressor if players perceive unsatisfactory game results as a threat to their global ranking ambitions (Hanton et al., 2005; Mellalieu et al., 2009). Stress caused by poor game performance might stimulate repeated cognitive activations (i.e., preservative cognitions), which can contribute to maintain the stress response (Brosschot et al., 2005, 2006). Importantly, stress related to game performance might disturb player's sleep (Fullagar et al., 2015). However, we still lack knowledge detailing the relation between esport players' performance and their subsequent sleep.

Sleep is an important tool for recovery and performance enhancements in any sport. However, sleep can be disturbed by stress (Eliasson and Vernalis, 2011), and esport players in particular are likely to suffer of suboptimal sleep because of their exposure to a range of other potential risk factors (e.g., coffeine use, competition events held in every global time zone, light exposure from screens, and lack of physical activity) (Bonnar et al., 2019; Lee et al., 2020). However, to the authors best knowledge, research that describes and investigates esport players' sleep patterns and distribution of the different sleep stages, is missing (Lee et al., 2021).

Sleep has an adaptive and restorative function both within the brain as well as the physiological processes in the body, and is considered the most valued recovery tool because of the consolidation and reconstruction of virtually all systems required for human development (Siegel, 2005; Stickgold, 2005; Venter, 2012; Nédélec et al., 2015; Bonnar et al., 2019). Sleep is divided into rapid-eye-movement sleep (REM) and non-REM sleep (NREM) (Carley and Farabi, 2016). REM sleep is found to be especially crucial for memory consolidation (Stickgold, 2005; Goldstein and Walker, 2014), and to prepare the esport players for emotional functioning the next day (Goldstein and Walker, 2014). REM sleep is also associated with increased activation in the primary motor cortex, which indicates that learning and motor memory are associated with this sleep stage (Walker et al., 2005; Nishida and Walker, 2007). REM sleep comprises normally about 20–25% of the total sleep time (Wagner et al., 2001). NREM sleep is divided into light sleep (N1 and N2) and deep sleep (N3, earlier N3 + N4) (Genzel et al., 2014). The light sleep stages comprise about 50–55% of total sleep time and are important for memory consolidation and sensory processing of external stimulus (Czisch et al., 2009). The deep sleep stage is the most restorative of all sleep stages, where brain waves, the respiratory system and muscle activity are at its lowest (Dijk, 2009). The N3 stage is especially important for physical restoration such as cardiovascular, muscular, and endocrine recovery (Dijk, 2009). More important for esport players is the function deep sleep has on learning and memory consolidation, especially declarative memory (Plihal and Born, 1997; Aeschbach et al., 2008; Spencer et al., 2017). The distribution of deep sleep normally makes up about 20–25% of the total sleep time. Thus, cognitive functioning, physiological processes, emotion regulation, and physical development are all associated with sleep (Hirshkowitz et al., 2015).

There is a decent line of research investigating associations between sleep and athletic performance in traditional sports (Lastella et al., 2014, 2015a,b; Juliff et al., 2015; O'Donnell et al., 2018) and researchers are encouraged to investigate such associations also in esport players (Bonnar et al., 2019). However, even in traditional sports the majority of the studies measure sleep by actigraphy, which is a wrist activity monitor, or by sleep diaries/questionnaires (Leeder et al., 2012). Neither of these measurements have sleep staging accuracy that is reliable and needed to explore associations between physical or psychological loads and recovery. Only a few of the studies measured sleep with polysomnography (PSG), which is the gold standard to detect sleep (Gupta et al., 2017). Importantly, research that includes esport players with longitudinal designs and measurements that detect sleep staging is needed to fully detect and describe their sleep patterns, and understand associations between their sleep and their performances and vice versa. Interestingly, a recent study among chess players indicated that sleep was associated with the players' performances (Moen et al., 2020). Chess players, like esport players, also depend on their perceptual cognitive skills to develop their performances. However, whether it is sleep that is predictive of performance, or performance that is predictive of sleep, is still unclear.

### The Present Study

It is conceivable that potential stress induced by poor game performances might negatively influence esport players' sleep (Morin et al., 2003; Fullagar et al., 2015). Based on this reasoning, it is hypothesized that better game performance will be associated with subsequent better sleep. To the authors knowledge, only a few recent studies have described sleep among esport players, but no study has investigated possible associations between players' game performances and players' subsequent sleep. The aims of the current study are thus two-fold: (1) to describe sleep patterns in esport players and their perceptions about their own sleep, and (2) to investigate prospective associations between game performance and sleep.

## Method

### Participants

Participants were recruited from a high school in Norway that offers esport 6 h per week as an educational program. There were 33 players who studied esport at the recruited school, and all of them were invited to participate in the current study. The esport players were given information about the research project at their digital e-learning platform, in which the importance, scope, and the data collection process were explained in detail. The information was given through the digital platform that the school uses. Because of the Covid-19 outbreak school was mainly organized with online digital solutions, and the players were expected to follow ordinary time schedules at school through the online digital solutions provided by their school during the period of data collection.

### Ethics Statement

REC Central, the Regional Committee for Medical and Health Research Ethics in Central Norway, founded on the Norwegian law on research ethics and medical research, has approved the study (project ID 2017/2072/REK midt).

### Procedure

Out of the 33 esport players who were invited to participate, 27 players decided to participate by signing the consent form approved by the local REC board and an agreement form for the use of the sleep monitoring equipment. Once all the players returned the signed consent forms, the necessary equipment for sleep monitoring was delivered, along with instructions for correct use. The equipment was disinfected and packed in a case 2 days before it was individually delivered to the players due to the Covid-19 pandemic. The players were instructed on the correct placement of the sleep monitor and the importance of correct settings for optimal functionality. The players received their equipment at least 7 days before the data collection started to ensure that everything worked properly, and to possibly have time to help the players to solve potential technical issues with the equipment. This was done to ensure that there were no technical issues with the equipment that could interfere with the data collection.

The players were then invited by mail to complete a survey that included questions about demographics such as age, sex, and subjective perceptions of their sleep. The data collection of sleep monitoring lasted for 56 days, from April the 13th to 7th of June, and entailed day-to-day monitoring of the players sleep patterns. All of the 27 players (24 males and 3 females, mean age 18.59 ± SD 2.80, range 17–32 years) completed the study and as a result, 1,512 data points with sleep could be collected. Researchers had access to real-time overview of each players' compliance with the study and monitored the progress closely throughout the whole period in order to solve any potentially technical issues that could occur in relation to the sleep monitoring systems. For objective sleep data, 1,243 (82%) of the potential maximum of 1,512 nights of sleep data were collected and analyzed. On average, 46.2 days were recorded for a player (SD = 7.1), with a minimum of 27 recorded days. Data was mainly lost due to technical issues with connecting the Somnofy units to wi-fi, especially when players were traveling away from their homes, such as to their cabins in weekends, and were unable to use the sleep monitoring device because of a poor wi-fi connection.

### Instruments

The instruments used in the current study were used to detect the esport players' subjective sleep perceptions, objective sleep and game performances.

#### Subjective Sleep

The players' perceptions of their sleep routines were measured with a single item for satisfaction about their sleep and if they struggled to fall asleep, using a 5-point Likert scale, ranging from 1 (never) to 5 (always): “*Do you feel that you get enough sleep?*” and “*Do you struggle to fall asleep when you go to bed?*”

#### Objective Sleep

The Somnofy sleep monitor is a novel, fully unobtrusive tool for sleep assessment, utilizing an impulse radio ultra-wideband (IR-UWB) pulse radar and Doppler technology. The IR-UWB radar emits radio wave pulses in the electromagnetic spectrum, which are able to pass through soft materials (i.e., clothes or duvets), but are reflected by denser materials (i.e., human body). As the pulses are reflected, they are returned and received by the IR-UWB radar again. Then, time-of-flight is used to analyze the time it takes to cover the distance between the radar to the object, and then back to the radar. The movement of the sleeping person and the person's respiration rate are derived from the IR-UWB radar by utilizing the Doppler effect and Fast Fourier Transform. In this way, Somnofy is able to monitor the vital signs, movement, and respiration, of the individual in bed with high precision with no attachments to the esport players' bodies. The raw data (movement and respiration) from the IR-UWB pulse radar are processed by a sleep algorithm, which uses machine learning to calculate relevant sleep variables. Recently, a full validation of Somnofy against manually scored Polysomnography (PSG) has confirmed Somnofy to be an adequate measure of sleep and wake, as well as sleep stages, in a healthy adult population (Toften et al., 2020). For the purposes of the current study, the following sleep variables were obtained from the Somnofy sleep monitor: sleep onset, sleep offset, time in bed, sleep onset latency, total sleep time, time in sleep stages (light, deep and REM), sleep efficiency, and respiration rate. A short description of the sleep variables that were measured in the study are shown in Table 1.

**Table 1:** Complete list of sleep variables derived from the sleep algorithm used in the sleep monitor.

**Sleep variable**	**Units**	**Characteristics of sleep variable**
Sleep onset	hh:mm	Time when sleep starts
Sleep offset	hh:mm	Time of wake-up
Time in bed	H	Total time spent in bed during the night
Sleep onset latency	H	The time it takes from when the athlete intends to go to sleep and actually starts to sleep
Total sleep time	H	Total sleep time obtained from sleep onset to time at wake-up
Deep sleep	h	Total amount of time in deep sleep (stage N3)
Light sleep	h	Total amount of time in light sleep (stage N1 and N2)
REM sleep	h	Total amount of time in REM sleep
Sleep efficiency	%	The percentage of time from sleep onset to wake-up time that was spent asleep
Respiration rate	Number	The number of respiratory ventilations per minute

*REM, rapid eye movement; NREM RPM, non-rapid eye movement respiration per minute*.

#### Game Performance

The players game performances are based on gaming statistics from games they performed in CS: GO. CS: GO is a multiplayer tactical first-person shooter (FPS) game. In FPS games the players control an avatar from a first-person view, which implies that the only visible thing on the screen is the hands and the weapons the avatars hold (Jonasson and Thiborg, 2010). CS: GO involves both tactical and precision pressure in a real-time setting (Schmidt et al., 2020), and provides a great diversity on its strategy skills, because it's played between two teams of five players each (Makarov et al., 2018). In CS: GO the players rank up in competitive matchmaking (Vaz, 2019), where an in-game ranking system sorts players into different groups based on their gaming skills (Poulus et al., 2020). The matchmaking in CS: GO is based on the Glicko-2 ranking system (Glickman, 2012), employing additional factors and modifications to adapt it to 5v5 scenarios (Vaz, 2019). Hence, players compete against random players from all over the world who are ranked on the same performance level.

The esport players were required to report at least 40 games during the period of data collection. For the purpose of the current study, gaming statistics from matches the players completed in CS: GO (matchmaking mode) were continually documented. A weekly game score was obtained by calculating the average kills minus deaths ratings from all the games they completed the current week. Thus, the weekly game score is the players calculated performance the current week, wherein higher score indicates better performance. A description of the detected game statistics and the calculated game scores are shown in Table 2.

**Table 2:** Variables detected from gaming statistics in CS: GO.

**Variable**	**Description**
Games wx	Total number of games completed week x
K wx	Total number of kills from all games completed week x
D wx	Total number of deaths from all games completed week x
Game score wx	(K wx-D wx)/Games wx = Weekly game score: measure of the esports players performance the current week

*wx, weekly average kills minus deats*.

### Statistical Analyses

**Figure 1:**
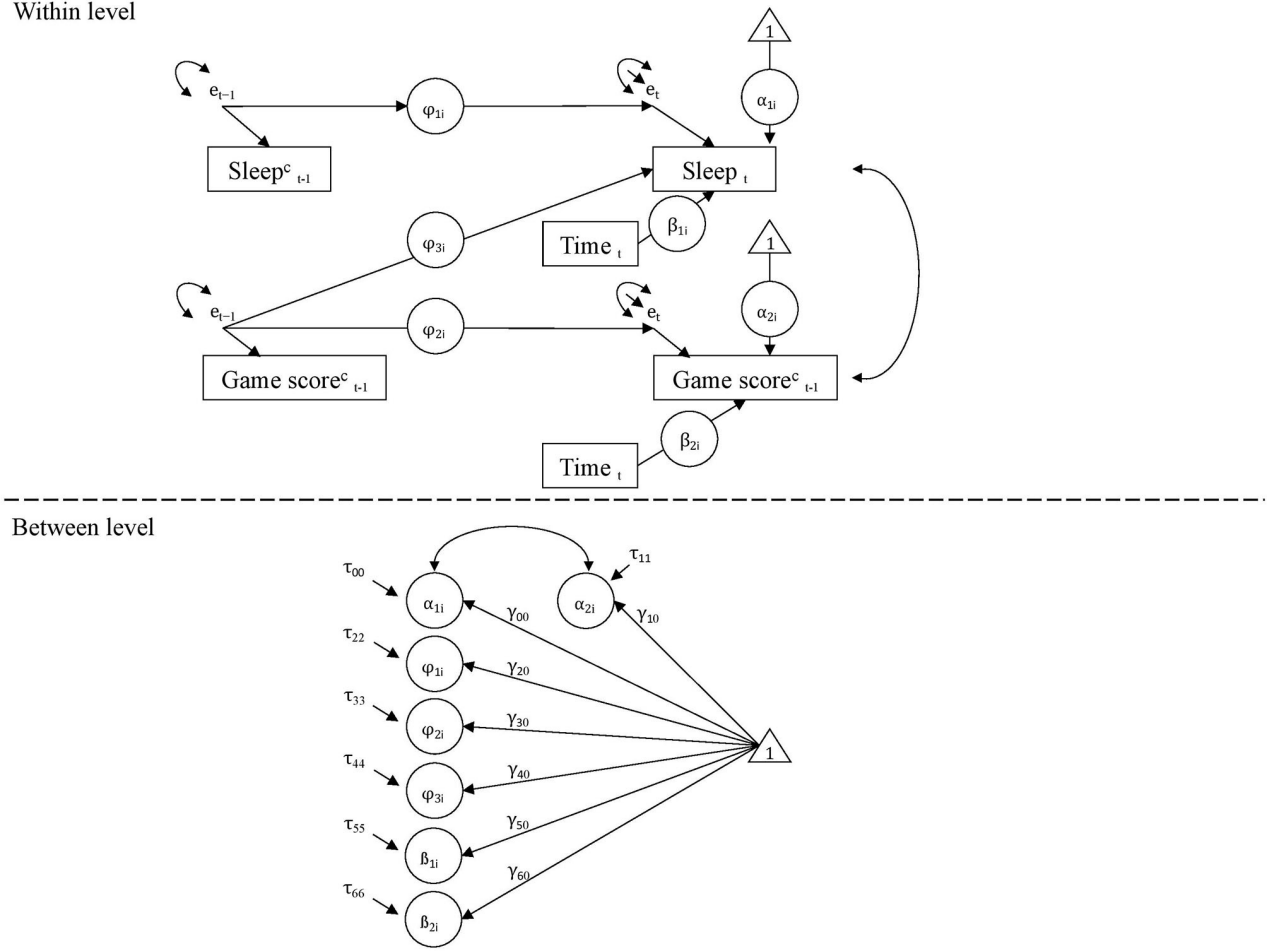
Conceptual diagram of the residual dynamic structural equation model (RDSEM).

IBM SPSS (version 25.0) was used to conduct demographic analyses and descriptive statistics of the players' sleep patterns. Intraclass correlations were computed in Mplus Version 8.1 (Muthén and Muthén, 2017). In order to examine whether game performance relates to successive sleep in longitudinal intensive data, residual dynamic structural equation modeling (RDSEM) was applied (McNeish and Hamaker, 2020). RDSEM combines features of multilevel modeling, structural equation modeling, time-series, and time-varying effects modeling (Asparouhov et al., 2018). In the employed RDSEM model, time-series measurements of sleep characteristics and game performance were partitioned into two levels: Level 1 encompasses within-person processes and Level 2 describes the between-person differences (McNeish and Hamaker, 2020). RDSEM employs residuals to estimate within-person autoregressive and cross-lagged estimates, while including time as a time-varying covariate. In addition to these Level 1 effects, RDSEM permits to examine the between-person associations between game performance and sleep (Level 2). The conceptual diagram of the employed model is illustrated in Figure 1.

RDSEM was run in Mplus 8.1, employing a Bayes full-information estimator with non-informative priors and no auxiliary variables, and using 2,000 Markov chain Monte Carlo iterations. Ten parallel models were fitted for game performance and each sleep characteristic separately. To account for within-person stability, daily sleep characteristic over a 56-days-period was regressed on sleep characteristic the preceding night (i.e., autoregression). Weekly game performance was also auto-regressed. Because sleep was measured daily, whereas game performance was assessed as a weekly average performance of the preceding 6 days, these 6 preceding days were coded as missing. Bayesian estimator uses all information available and provides estimates unbiased by missingness (Muthén and Asparouhov, 2012; Schuurman et al., 2016; Asparouhov and Muthén, 2020). In each model, within-person cross-lagged paths were estimated from game performance in the preceding week predicting subsequent sleep characteristic. To account for the concurrent associations between sleep and game performance, a covariance between these measures was estimated at Level 1. At the between-person Level 2, a covariance between mean levels of sleep and game performance was estimated. Random effects (i.e., between-person variances) of Level 1 paths (i.e., autoregressive and cross-lagged effects) were estimated at Level 2. To account for temporal trends, the estimates were portioned out from linear trends for each person (Curran and Bauer, 2011).

## Results

### Subjective Perceptions of Sleep and Descriptive Statistics

The players' mean score of their subjective sleep satisfaction was 3.37 (SD = 0.63), and their perception of their struggless to fall asleep was 3.15 (SD = 0.95), indicating that the esport players in the current study were not satisfied with their sleep and their abilities to fall asleep. Table 3 shows statistics about the players' game performance during the period of data collection.

**Table 3:** Descriptive statistics for the gaming performances based on 8 weeks of gaming data in 27 esport players.

**Gaming variable**	**N**	**Min**	**Max**	**Mean**	**SD**
Game socre w1	26	−7.20	12.00	3.49	5.03
Game score w2	26	−5.80	15.33	2.79	5.16
Game score w3	27	−6.50	15.38	3.40	4.70
Game score w4	27	−8.40	11.00	3.08	4.62
Game score w5	27	−6.00	12.40	2.71	4.22
Game score w6	27	−5.00	10.00	1.83	4.16
Game score w7	25	−7.60	11.60	1.58	4.82
Game score w8	26	−6.33	12.20	2.69	4.25

*Game score wx, weekly game score week x*.

The esport players completed in average 5.77 games pr. week, ranging from 0 to 19 games. During the period of data collection the esport players completed on average 46.15 games, ranging from 40 to 67 games. Thus, the data collection resulted in a total number of 1,246 reported games that were collected and analyzed. Descriptive statistics of the esport players' sleep patterns are shown in Table 4.

**Table 4:** Descriptive statistics for the objective sleep patterns, based on 1243 nights of data in 27 esport players.

**Sleep variable**	**Mean**	**STD**
Sleep onset (hh:mm)	02:09	02:01
Sleep offset (hh:mm)	10:10	02:32
Time in bed (h)	10:58	03:32
Sleep onset latency (h)	00:57	00:54
Total sleep time (h)	07:12	01:54
Light sleep (h)/(%)	04:01/55.1	01:17/7.5
Deep sleep (h)/(%)	01:23/20.0	00:25/6.6
REM sleep (h)/(%)	01:45/24.9	00:40/6.3
Sleep efficiency (%)	67.7	16.0
NREM RPM (N)	14.75	2.2

*STD, standard deviation; REM, rapid eye movement; NREM RPM, non-rapid eye movement respiration per minute*.

The average sleep onset and sleep offset in esport players were 02:09 and 10:01, respectively, and the average total sleep time was 07:12 h per night.

### Interclass Correlations

The authors first assessed how much variance in sleep could be attributed to grouping within a person. NREM RPM sleep, sleep efficiency, time in bed, sleep onset and offset showed that a considerable proportion of the variation could be attributed to individuals (ranging from 0.77 to 0.29; Table 5). In contrast, REM, light and deep sleep, sleep time, and sleep onset latency did not vary much between individuals, but rather between measurement occassions.The fitted RDSEM models explained between 15% (deep sleep) and 42% (NREM RPM) of the within-person variability in sleep characteristics.

**Table 5:** Unstandardized and standardized estimates.

		**Sleep onset**				**Sleep offset**				**Time in bed**			
**ICC**		**0.40**				**0.29**				**0.41**			
**DIC**		**14066.752**				**15676.156**				**15449.446**			
		**Un-stand. est**.	**95 % CI**	**Stand. est**.	**95 % CI**	**Un-stand. est**.	**95 % CI**	**Stand. est**.	**95 % CI**	**Un-stand. est**.	**95 % CI**	**Stand. est**.	**95 % CI**
Correlation sleep with game score on the between level	*r*	−0.12	−4.07 to 4.07	−0.02	−0.45 to 0.42	1.63	−2.05 to 6.67	0.23	−0.26 to 0.64	4.79	−0.44 to 14.16	0.40	−0.04 to 0.72
**Means**													
Sleep (μ Sleep)	γ_00_	26.20	25.56 to 26.94			10.54	9.79 – 11.38			11.09	10.06 to 12.24		
Game score	γ_10_	3.16	1.13 to 5.16			3.74	1.86 – 5.51			3.67	1.69 – 5.77		
**Slope**													
Game → Sleep	γ_40_	−0.06	−0.21 to 0.09	−0.09	−0.23 to 0.01	−0.22	−0.41 to 0.004	−0.21	−0.36 to 0.09	−0.28	−0.61 to 0.08	−0.16	−0.27 to 0.02
**Autoregression**													
Sleep	γ_20_	0.20	0.08 to 0.31	0.20	0.10 to 0.29	0.02	0.004 to 0.07	0.09	0.002 to 0.18	0.13	0.01 to 0.24	0.13	0.03 to 0.23
Game score	γ_30_	−0.002	−0.22 to 0.23	−0.004	−0.20 to 0.20	0.07	−0.14 to 0.27	0.07	−0.13 to 0.25	−0.08	−0.29 to 0.14	−0.08	−0.27 to 0.12
**Variances**													
Sleep	τ_00_	2.74	1.41 to 5.68			3.50	1.80 – 7.35			7.09	3.45 to 14.56		
Game score	τ_11_	19.37	9.61 to 42. 71			16.00	7.68 to 34.98			22.50	11.41 to 48.27		
Game → Sleep	τ_44_	0.11				0.19	0.09 to 0.40			0.64	0.33 to 1.29		
*R* ^2^		0.38	0.32 to 0.44			0.30	0.24 to 0.37			0.39	0.33 to 0.46		
		**Sleep onset latency** ^**a**^				**Total sleep time**				**Deep Sleep** ^**b**^			
ICC		0.14				0.06				0.11			
DIC		7863.043				14812.543				10129.128			
		**Un-stand. est**	**95% CI**	**Stand. est**.	**95% CI**	**Un-stand. est**	**95% CI**	**Stand. est**	**95% CI**	**Un-stand. est**	**95% CI**	**Stand. est**	**95% CI**
Correlation sleep with game score on the between level	*r*	−0.10	−0.30 to 0.04	−0.34	−0.72 to 0.15	1.14	0.03 to 3.20	0.55	0.01 to 0.86	0.10	−0.24 to 0.54	0.18	−0.35 to 0.63
**Means**													
Sleep (μ Sleep)	γ_00_	0.94	0.79 to 1.10			7.28	6.99 to 7.60			1.40	1.33 to 1.48		
Game score	γ_10_	0.01	−0.33 to 0.34			3.35	1.40 to 5.28			3.73	1.85 to 5.48		
**Slope**													
Game → Sleep	γ_40_	–	–	–	–	−0.06	−0.22 to 0.10	−0.06	−0.22 to 0.08	0.03	−0.004 to 0.05	0.20	0.05 to 0.33
**Autoregression**													
Sleep	γ_20_	0.03	−0.06 to 0.13	0.03	−0.05 to 0.10	−0.006	−0.10 to 0.10	−0.01	−0.09 to 0.08	0.01	−0.08 to 0.10	0.01	−0.07 to 0.10
Game score	γ_30_	0.03	−0.06 to 0.13	0.03	−0.05 to 0.10	−0.11	−0.35 to 0.06	−0.10	−0.35 to 0.05	0.13	−0.25 to 0.54	0.12	−0.23 to 0.53
**Variances**													
Sleep	τ_00_	0.13	0.07 to 0.27			0.29	0.09 to 0.79			0.02	0.01 to 0.05		
Game score	τ_11_	0.66	0.25 to 1.36			17.08	8.92 to 38.03			15.98	7.94 to 34.92		
Game → Sleep	τ_44_	–	–			0.12	0.06 to 0.24			0.002	0.001 to 0.006		
*R* ^2^		0.06	0.03 to 0.12			0.25	0.18 to 0.32			0.15	0.08 to 0.21		
		**Light sleep**				**REM sleep**				**Sleep efficiency** ^**c**^			
ICC		0.10				0.06				0.69			
DIC		13483.111				11815.578				20697.662			
		**Un-stand. est**.	**95 % CI**	**Stand. est**.	**95 % CI**	**Un-stand. est**.	**95 % CI**	**Stand. est**.	**95 % CI**	**Un- stand. est**.	**95 % CI**	**Stand. est**.	**95 % CI**
Correlation sleep with game score on the between level	*r*	0.75	−0.26 to 2.38	0.42	−0.13 to 0.77	0.32	−0.03 to 0.96	0.48	−0.04 to 0.84	1.31	−0.21.56 to 25.13	0.03	−0.43 to 0.48
**Means**													
Sleep (μ Sleep)	γ_00_	4.10	3.88 to 4.34			1.76	1.66 to 1.87			66.66	62.19 to 71.15		
Game score	γ_10_	3.29	1.36 to 5.16			3.23	1.39 to 5.08			3.51	1.67 to 5.39		
**Slope**													
Game → Sleep	γ_40_	−0.07	−0.03 to 0.17	−0.14	−0.26 to 0.004	−0.03	−0.08 to 0.03	−0.10	−0.24 to 0.04	0.30	−0.70 to 1.35	0.04	−0.10 to 0.16
**Autoregression**													
Sleep	γ_20_	0.07	−0.03 to 0.17	0.07	−0.02 to 0.16	−0.05	−0.12 to 0.04	−0.05	−0.12 to 0.03	0.11	0.01 to 0.21	0.10	0.02 to 0.19
Game score	γ_30_	−0.15	−0.39 to 0.10	−0.15	−0.38 to 0.08	−0.08	−0.30 to 0.12	−0.08	−0.29 to 0.11	−0.02	−0.32 to 0.29	0.01	−0.30 to 0.31
**Variances**													
Sleep	τ_00_	0.21	0.08 to 0 0.48			0.03	0.01 to 0.08			115.07	61.57 to 232.99		
Game score	τ_11_	18.10	9.10 to 40.28			16.16	7.90 to 36.24			15.12	7.43 to 33.03		
Game → Sleep	τ_44_	0.05	0.03 to 0.11			0.01	0.01 to 0.02			3.43	1.39 to 7.68		
*R* ^2^		0.26	0.20 to 0.32			0.22	0.16 to 0.28			0.19	0.11 to 0.26		
				**NREM RPM**									
ICC		0.77											
DIC		11556.281											
		**Un-stand. est**.	**95 % CI**	**Stand. est**.	**95 % CI**								
Correlation sleep with game score on the between level	*r*	−3.86	−0.10.46 to 0.11	−0.44	−0.75 to 0.02								
**Means**													
Sleep (μ Sleep)	γ_00_	14.60	13.78 – 15.48										
Game score	γ_10_	3.12	1.08 – 5.05										
**Slope**													
Game → Sleep	γ_40_	−0.07	−0.14 to 0.003	−0.21	−0.33 to 0.06								
**Autoregression**													
Sleep	γ_20_	0.25	0.13 to 0.36	0.25	0.16 to 0.33								
Game score	γ_30_	0.05	−0.13 to 0.20	0.05	−0.10 to 0.19								
**Variances**													
Sleep	τ_00_	4.42	2.57 to 8.64										
Game score	τ_11_	18.15	8.48 to 40.22										
Game → Sleep	τ_44_	0.03	0.02 to 0.06										
R^2^		0.42	0.37 to 0.47										

*^a^In order to reach convergence, this model was fit slightly differently: timepoint and game score were standardized, and several random effects were exluded from the analyzes (including the cross-lagged slope). *

*^b^Five clusters were removed.*

*^c^Three clusters were removed.*

*R^2^, explained variance in sleep; ICC, intraclass correlation; DIC, Deviance information criteria; CI, Credible interval; Un-stand. est., unstandardized estimate; Stand. est., standardized estimate*.

### Unstandardized Within-Level Autoregression and Cross-Lagged Estimates, and Between-Level Correlations

The unstandardized autoregressive values for sleep characteristics were significant for some of the sleep measures (i.e., NREM RPM 0.25, sleep onset 0.20, time in bed 0.13, sleep efficiency 0.11, and sleep offset 0.02) (Table 5). These estimates suggest that after a night with rather high or low scores on these sleep characteristics, there is a rather weak tendency to stay high or low on these characteristics, before returning to individual's typical set point. Other sleep characteristics' (i.e., sleep onset latency, total sleep time, light sleep, deep sleep, and REM sleep) autoregressive effects turned non-significant, suggesting a strong attraction to individual's typical values after a night with high og low values on their sleep characteristics. Similarly, there was no significant carry-over effect of preceding game performance on successive game performance in this period.

The cross-lagged unstandardized values revealed that, on average and across individuals, weekly game performance did not predict any of the subsequent sleep measures, with one exception: game performance was a significant predictor of sleep offset (−0.22, 95% credible interval −0.41 to −0.004; Table 5). This suggests that individuals who scored higher on their game performance compared to their personal mean would wake up earlier the following day. There was also a considerable variance around this slope (0.19, with 95% credible interval 0.09–0.40), suggesting that individuals vary in the extent to how much their performance influences their wake-up time.

On the between-level, individuals with better game performance spent more time sleeping (total sleep time, *r* = 0.55) and scored lower on NREM RPM (*r* = −0.44).

### Standardized Autoregression and Cross-Lagged Estimates

The within-level standardized estimates averaged over clusters (Schuurman et al., 2016) cannot be used as an inference about a hypothetical population in the same way as the unstandardized results do, but merely describe associations in the present sample and enable effects comparison across different measures. As such, the highest carry-over or stability in sleep characteristics was seen in NREM RPM (0.25) and sleep onset (0.20), whereas the autoregression estimates were smaller in respect to time in bed (0.13), sleep efficiency (0.04), and sleep offset (0.09) (Table 5).

The within-level standardized cross-lagged effect of game performance on sleep was largest in respect to sleep offset (−0.21) and NREM RPM (−0.21), but it turned-out significant also for other sleep measures (i.e., deep sleep 0.20, time in bed −0.16, light sleep (−0.14, and sleep onset −0.09). These results revealed that in the current sample, esport players who scored higher in their game performance in the preceding week spent subsequently more time in deep sleep, less time in light sleep and had lower NREM RPM, less time in bed, woke up earlier, and also fell asleep somewhat earlier, compared to their typical personal-specific values.

## Discussion

The aim of the current study was to examine sleep characteristics and subjective sleep perceptions in a sample of 27 esport players, and to investigate how their game performances were associated with their sleep. To the authors knowledge there are currently no studies that document sleep characteristics among esport players with sleep staging. The hypothesis of the current study predicted that the esport players game performances were associated with their sleep. The prediction was partly confirmed. The results from the within-person unstandardized values showed that the players individual game performances were associated only with sleep offset. However, the sample-specific within-level standardized values showed that the individual game performances of the players were associated with their sleep, wherein their game performance was a significant predictor of sleep onset, sleep offset, deep sleep, time in bed, and light sleep. In addition, on the group level, the esport players who performed better also displayed significantly more time in total sleep time and lower NREM RPM.

### Sleep Characteristics and Subjective Perceptions of Esport Players

The results of the current study showed that the esport players obtained a mean of 07:12 h (SD = 01:54) of total sleep per night during the 56 days of sleep monitoring, and that they fell asleep late at night (02:09) and woke up late in the morning (10:10). The amount of sleep that was detected is at the minimum level of sleep recommendations for the general population (7–9 h) in this age group (Hirshkowitz et al., 2015) and below the recommendations (9–10 h) for athletes in traditional sports (Bird, 2013; Watson, 2017). While some studies among athletes in different traditional sports reported longer sleep durations (Gupta et al., 2017; Hrozanova et al., 2018), others also detected shorter sleep durations (Leeder et al., 2012). A recent study in a comparable sport such as chess and within the same age group reported shorter sleep time (6:44) (Moen et al., 2020). A recent study among professional esport players showed a median total sleep time of 6:48 h per night over a period of 7–14 days (Lee et al., 2021), and another study reported median total sleep time 7:26 h per night (Lee et al., 2020). Thus, the total sleep time among esport players seems to be 7 h per night ±15 min.

However, the results of the current study showed that the esport players were fairly dissatisfied with their sleep (mean = 3.37, max 5) and that they also struggled to fall asleep at night (mean = 3.15, max 5). Their experienced difficulties to fall asleep when they intended to fall asleep were supported by sleep onset latency (SOL), wherein esport players used 0:57 h (SD = 0:54) to fall asleep. The SOL time is also higher compared to findings among chess players in the same age group (Moen et al., 2020). The current results showed later sleep onset and sleep offset than what is normal among athletes in other traditional sports (Hrozanova et al., 2020). However, the results in the current study confirm results from a recent study among esport players that show both delayed sleep patterns and prolonged wake after sleep onset (Lee et al., 2021). A potential explanation might be that games are played among competitors in every global time zone, and hence also at night time.

Sleep efficiency among the esport players in the current study was 67.7% (SD = 16), which is significantly below the recommended levels (>85%) and below what is found among athletes in different sports (Leeder et al., 2012; Gupta et al., 2017; Ohayon et al., 2017; Hrozanova et al., 2018; Moen et al., 2020), or among other esport players (Lee et al., 2021). The poor sleep efficiency in the current study might have determinable effects on the esport players, because poor sleep efficiency is associated with impaired daytime functioning (Kirmil-Gray et al., 1984). Nevertheless, the distribution of the players' sleep into sleep stages is within the recommended levels, wherein 55.1% (SD = 7.5) of their sleep was spent in light sleep, 20% (SD = 6.6) in deep sleep and 24.9% (SD = 6.3) in REM sleep. The results also showed that there were considerable individual differences between the players and that their individual sleep patterns were quite consistent.

In sum, the sleep characteristics that are found in the current study indicate that the players' sleep was not optimal. A potential explanation of the esport players' sleep characteristics conveyed in the current study might be exposure to blue light from gaming devices. Blue light from digital devices is found to influence sleep negatively (Chang et al., 2015; Green et al., 2017). Exposure of blue light from digital devices can influence the circadian timing system. Especially exposure late in the evening is associated with prolonged sleep onset latency (Chang et al., 2015; Green et al., 2017) and research claims that exposure from blue light from digital devices might have a negative impact on adolescents sleep efficiency (Fobian et al., 2016). Interestingly, total sleep time extending beyond recommendations has been associated with improved athletic performances within shooting, sprint, and reaction (Mah et al., 2011), and some research suggests that extended sleep leads to improvements in daytime alertness and mood (Hirshkowitz et al., 2015). The documented benefits of extended sleep can be relevant also for the esport players, as extended sleep might enhance their performances.

### Individual Sleep Patterns

The results of the current study also showed that the individual sleep patterns among the esport players vary within an individual pattern and that there are large individual differences among the players (based om standard deviation values). The significant ICC and autoregressive values (Table 5) show that there are individual stable variations with respect to sleep onset, sleep offset, time in bed, sleep effiency, and NREM RPM. Thus, the results indicate that the players have stable sleep patterns regarding when they fall asleep and wake up, how much time they spend in their beds and their sleep efficiency. Their NREM respiration rates are also stable. The current results indicate that these sleep patterns are stable within individual players, which is in line with recent findings (Costa et al., 2019).

Interestingly, deep sleep, REM sleep, light sleep and total sleep time were not found to have the same individual stable variation. Importantly, it is expected that the distribution of these sleep stages varies within individuals, since the different sleep stages have different tasks in the recovery process as we have outlined in the introduction, and that recovery demands will vary regarding variations in the stress loads the players are exposed to (Moen et al., 2020).

Our findings thereby suggest that the sleep hygiene among the esport players seems to be individual and stable, as the players seem to fall asleep, wake up and stay in bed in the same routinely time pattern. However, the sleep stages seem to display an individual unstable variation which might differ from day to day. These outlined differences in patterns of sleep hygiene and sleep stages underscore the importance of analyzing both the distribution of sleep stages as well as sleep routine patterns when examining sleep.

### Associations Between Game Performances and Sleep

The results from the RDSEM analyses on within level showed that the esport players' weekly game performances significantly predicted their sleep offset in the following week. In addition, the sample specific estimates (standardized values) showed that higher game performance scores predicted significantly earlier sleep onset, earlier sleep offset, more time in deep sleep, less time in light sleep, and lower NREM respiration rate values. Further, the results from the RDSEM on the between level showed that the esport players who had performed better also displayed significantly more time in total sleep and significantly lower NREM respiration values than the esport players who didn't score as well on their game performances. Thus, the results of the current study give reason to discuss why better game performances seem to be predictive of a more favorable sleep pattern.

First, the strong significant correlations between game performance and non-REM respiration rate (−0.44), and game performance and total sleep time (0.55) might indicate that better game performances might lead to less perceived negative stress (Pedraza-Ramirez et al., 2020). On the contrary, if game demands exceed players' capabilities, the players might failure to meet their performance expectation and a negative stress response might be activated. This negative response may be caused by both their individual expectations and by expectations of their team members, as expressed through in-game communication, critisicm and negative evaluations (Smith et al., 2019). Because stress is a common predictor of sleep disturbance (Eliasson and Vernalis, 2011), such a negative response elicitated by poor game performance might result in less sleep. This line of reasoning would align with our finding that players who perform better also sleep more. Importantly, research suggests that the respiratory ventilation of the body will increase when humans are faced with internal or external stressors (Tipton et al., 2017). Such evidence connectiong stress and rates of respiratory ventilation aligns well with the current finding of lower non-REM respiration rate among players with better game performance. Interestingly, a recent study also suggested that less favorable sleep patterns documented among chess players that experienced negative performance development over a period of 4 months, could be explained by their experiences of distress (Moen et al., 2020). A recent study among junior athletes has also shown that worry and perceived stress were negatively associated with sleep quality (Hrozanova et al., 2019).

The conveyed association between game performance of the players and their NREM respiration rates and the suggested associations to stress, might also serve as a potential explanation of the sample-specific findings connecting game performances to earlier sleep onset, more time in deep sleep, less time in light sleep and earlier sleep offset. When players experience high levels of stress they are stimulated to engage in repeated engagement in cognitive activations such as worrying, rumination, and cognitive arousals (Wells, 2009). Accordingly, esport players who experience positive performance development experience less negative stress, which in turn might result in fewer negative thoughts and emotions before bedtime (Morin et al., 2003). Such cognitive activations have the potential to keep the stress-related content from resolving and cause the stress response systems of the body to remain activated (Brosschot et al., 2005). Thus, both cognitive activations and potential remained stress activity make it difficult to fall asleep earlier at night, which aligns with the current findings of better game performances predicting earlier sleep onset and offset. Further, players who perform better might go to bed in a more relaxed state, which makes it easier for the body to obtain more time in deep sleep and less time in light sleep during the sleep cycles throughout the night. This again aligns with the current results showing that better game performances are predictive of more time in deep sleep and less time in light sleep. Finally, when esport players spend more time in deep sleep where the respiratory system and muscle activity are at its lowest during sleep (Dijk, 2009), they might recover better and wake up earlier as a consequence. Such line of reasoning might explain the unstandardized results showing better game performances to be predictive of earlier sleep offset. In sum, the findings of the current study suggest that better game performances is linked to better sleep.

### Strengths and Limitations

The strengths of the current study encompass longitudinal intensive data of game performance and objectively measured sleep assessment in esport players. Nevertheless, there are several limitations that should be kept in mind when interpreting the results. First of all, the present results should be interpreted within the context of the Covid-19 pandemic that took place during the period of data collection. Secondly, the number of participants was rather low, and low number of participants influence the power to find significant associations in the multilevel statistical analyses (RDSEM) that were used in the current study. In addition, game performance was observed weekly (8) per participant, and not daily as was the case for the sleep variables (56), thereby possible influencing the power of the RDSEM analyses. Thirdly, there are other relevant variables that may influence the players' sleep patterns such as physical and psychological daily stress loads, that were not included in the present study. Also, 18% of all potential data was lost mainly because of difficulties connected to the local wi-fi network, especially when the players stayed at different locations outside their homes. Finally, the study did not control for sleep during daytime and napping frequency (Petit et al., 2014). Potential interpretations of the current results must take these limitations into consideration.

## Conclusion

The results of the current study provide valuable insight into sleep characteristics and sleep patterns among esport players, and also insight into how the esport players' game performance are related to their subsequent sleep. Better performing players slept more and they scored lower on non-REM respiration rates. Even though the unstandardized results revealed that better performance was related only to earlier sleep offset, the sample-specific results suggested that better game performances might enhance several measures of players' sleep. It is suggested that negative response elicitated by poor game performance might explain these findings.

## Data Availability Statement

The raw data supporting the conclusions of this article will be made available by the authors, without undue reservation.

## Author Contributions

FM, MV, VS, and JH contributed to the conception and design of the study. VS, FM, and MV performed the statistical analysis. FM and MV wrote the first draft of the manuscript. MO, FM, and MV organized the database. VS wrote sections of the manuscript. All authors contributed to the manuscript revision, read, and approved the submitted version.

## Funding

This study was funded by the Center for Elite Sports Research, Department of Neuromedicine and Movement Science and Department of Sociology and Political Science, at the Norwegian University of Science and Technology, Trondheim, Norway.

## Conflict of Interest

The authors declare that the research was conducted in the absence of any commercial or financial relationships that could be construed as a potential conflict of interest.

## Publisher's Note

All claims expressed in this article are solely those of the authors and do not necessarily represent those of their affiliated organizations, or those of the publisher, the editors and the reviewers. Any product that may be evaluated in this article, or claim that may be made by its manufacturer, is not guaranteed or endorsed by the publisher.
